# Comparative clinical characteristics and outcomes of patients with community acquired bacteremia caused by *Escherichia coli*, *Burkholderia pseudomallei* and *Staphylococcus aureus*: A prospective observational study (Ubon-sepsis)

**DOI:** 10.1371/journal.pntd.0009704

**Published:** 2021-09-03

**Authors:** Ranjani Somayaji, Viriya Hantrakun, Prapit Teparrukkul, Gumphol Wongsuvan, Kristina E. Rudd, Nicholas P. J. Day, T. Eoin West, Direk Limmathurotsakul

**Affiliations:** 1 Department of Medicine, University of Calgary, Calgary, Alberta, Canada; 2 Mahidol Oxford Tropical Medicine Research Unit, Faculty of Tropical Medicine, Mahidol University, Bangkok, Thailand; 3 Department of Internal Medicine, Sunpasitthiprasong Hospital, Ubon Ratchathani, Thailand; 4 Department of Critical Care Medicine, University of Pittsburgh, Pittsburgh, Pennsylvania, United States of America; 5 Centre for Tropical Medicine and Global Health, Nuffield Department of Medicine, University of Oxford, Churchill Hospital, Oxford, United Kingdom; 6 Department of Pulmonology, Critical Care and Sleep Medicine, University of Washington, Seattle, Washington, United States of America; 7 Department of Microbiology and Immunology, Faculty of Tropical Medicine, Mahidol University, Bangkok, Thailand; 8 Department of Tropical Hygiene, Faculty of Tropical Medicine, Mahidol University, Bangkok, Thailand; Aga Khan University Hospital, PAKISTAN

## Abstract

**Background:**

Community acquired bacteremia (CAB) is a common cause of sepsis in low and middle-income countries (LMICs). However, knowledge about factors associated with outcomes of CAB in LMICs is limited.

**Methodology/Principal findings:**

A prospective observational study (Ubon-sepsis) of adults admitted to a referral hospital with community-acquired infection in Northeastern Thailand was conducted between March 1, 2013 and February 1, 2017. In the present analysis, patients with a blood culture collected within 24 hours of admission that was positive for one of the three most common pathogens were studied. Clinical features, management, and outcomes of patients with each cause of CAB were compared. Of 3,806 patients presenting with community-acquired sepsis, 155, 131 and 37 patients had a blood culture positive for *Escherichia coli*, *Burkholderia pseudomallei* and *Staphylococcus aureus*, respectively. Of these 323 CAB patients, 284 (89%) were transferred from other hospitals. 28-day mortality was highest in patients with *B*. *pseudomallei* bactaeremia (66%), followed by those with *S*. *aureus* bacteraemia (43%) and *E*. *coli* (19%) bacteraemia. In the multivariable Cox proportional hazards model adjusted for age, sex, transfer from another hospital, empirical antibiotics prior to or during the transfer, and presence of organ dysfunction on admission, *B*. *pseudomallei* (aHR 3.78; 95%CI 2.31–6.21) and *S*. *aureus* (aHR 2.72; 95%CI 1.40–5.28) bacteraemias were associated with higher mortality compared to *E*. *coli* bacteraemia. Receiving empirical antibiotics recommended for CAB caused by the etiologic organism prior to or during transfer was associated with survival (aHR 0.58; 95%CI 0.38–0.88).

**Conclusions/Significance:**

Mortality of patients with CAB caused by *B*. *pseudomallei* was higher than those caused by *S*. *aureus* and *E*. *coli*, even after adjusting for presence of organ dysfunction on admission and effectiveness of empirical antibiotics received. Improving algorithms or rapid diagnostic tests to guide early empirical antibiotic may be key to improving CAB outcomes in LMICs.

## Author summary

Bloodstream infections are very common and associated with significant numbers of death and disability around the world. Less is known about the rates, risks and impact of common bloodstream infections in low and middle-income countries. We conducted a large prospective four-year observational study in northeastern Thailand of persons admitted to a tertiary-care hospital with severe infections and analyzed those who had bloodstream infections. The three most common bacteria causing bloodstream infections were *Escherichia coli*, *Burkholderia pseudomallei* and *Staphylococcus aureus*. Most of the patients were transferred from other hospitals. Overall mortality was 41%. The highest mortality was among those who had *B*. *pseudomallei* infection, at 66%, and the lowest mortality was among those who had *E*. *coli* infection, at 19%. If appropriate antibiotics were given early, i.e. prior to or during the transfer, this was associated with survival. Our large study was able to identify the types, risk factors and impact of bloodstream infections in a low and middle-income country. These important results highlight the need for the development and implementation of rapid diagnostic tests and hospital algorithms for early diagnosis to improve care and outcomes of bloodstream infections in low and middle-income countries.

## Background

Community-acquired bacteremia (CAB) or bloodstream infection is a common cause of sepsis, potentially leading to death, in low and middle-income countries (LMICs) [1, 2]. Sepsis is a life-threatening organ dysfunction due to dysregulated host response to infection [1]. In 2017, 11 million sepsis-related deaths were estimated to occur worldwide, with the highest burden in sub-Saharan Africa, Oceania, south Asia, east Asia and southeast Asia [3]. The importance of obtaining a blood culture especially in persons with sepsis has been highlighted in sepsis guidelines to identify the cause of infection and ensure appropriate antimicrobial therapies [2]. In high-income countries, the rate of hospitalization due to CAB is around 77 to 92 per 100,000 people per year, and the 30-day mortality is around 13 to 19% [4–8]. However, due to challenges of access to diagnostic microbiology facilities and data sources, our understanding of the epidemiology and outcomes of CAB in resource-limited settings remains rudimentary [9, 10].

In recent years, there have been greater efforts to examine the clinical characteristics and deaths of patients with bacteremia caused by specific pathogens in tropical developing countries and particularly in South-east Asia [9]. Nonetheless, the need for comprehensive studies to improve our understanding and to develop antibiotic prescribing recommendations for CAB or community-acquired sepsis cannot be understated. Observational studies in Northeast Thailand have demonstrated a rising incidence of CAB with a significant all-cause mortality [11–15]. The most commonly identified organisms causing CAB in this region are *Escherichia coli*, *Burkholderia pseudomallei*, and *Staphylococcus aureus* [11]. While *E*. *coli*, *B*. *pseudomallei* and *S*. *aureus* bacteremia have each been studied in isolation [9, 12–15], no studies to date have prospectively and directly compared these bacteremia which may provide discrete or overlapping risk factors and epidemiology to utilize in screening and treatment pathways. Thus, we sought to evaluate clinical characteristics, treatment and outcomes of hospitalized patients with CAB caused by *E*. *coli*, *B*. *pseudomallei*, or *S*. *aureus* in Northeast Thailand, a representative resource-limited setting.

## Methods

### Ethics statement

The study was approved by the ethics committees at Sunpasitthiprasong Hospital (039/2556), Mahidol University Faculty of Tropical Medicine (MUTM2012-024-01), the University of Washington IRB (42988) and the University of Oxford (OXTREC172-12). Informed consent was obtained from all study participants.

### Study design and study site

This was a pre-planned analysis of the Ubon-Sepsis study, a prospective observational study completed in Sunpasitthiprasong Hospital in Ubon Ratchathani province, Northeast Thailand from March 1, 2013 through February 1, 2017 (NCT02217592); the details of the study design and variables are described elsewhere [16–18]. Ubon Ratchathani has a population of 1.8 million persons and is the largest province with an area of 16,113 km^2^. The study site is a public tertiary-care hospital and acts as a referral center due to its availability of intensive care unit (ICU) and microbiology facilities.

### Study participants

The study prospectively enrolled consenting patients ≥18 years old who were admitted to the general medical wards or the medical ICUs with a primary diagnosis of community-acquired infection made by the attending physician, were within 24 hours of admission to the study hospital, and had at least three Surviving Sepsis Campaign criteria for sepsis documented in the medical record (S1 Table from primary study publication) [16]. Patients with a previous hospitalization within the past 30 days, those who were transferred from other hospitals with a total duration of hospitalization >72 hours and those who were diagnosed with hospital-acquired infections were excluded. In this study, patients with blood culture collected within 24 hours of admission culture positive for the most common three pathogens were included for analysis.

### Study team point-of-care assessments

Following enrollment, patients were evaluated by the study nurses using four point-of-care assessments; including a whole blood glucose rapid diagnostic test (RDT) (ACCU-CHECK Performa, Roche Diagnostic, Germany), a whole blood lactate RDT (Lactate Pro 2, Arkray Global Business Inc., Australia), pulse oximetry (Nellcor N-65, Covidien plc., Ireland) and the Glasgow Coma Scale (GCS). Blood samples (4±10 mL) were also collected for culture using BD BACTEC automated blood culture system (Becton-Dickinson, Sparks, MD, USA) [16]. During the study period, antimicrobial susceptibility was determined using the disc diffusion method according to Clinical and Laboratory Standards Institute (CLSI) [19]. The results were reported to the attending physicians. The study did not involve any clinical interventions and all medical treatment was provided by the attending physicians and respective medical teams.

### Data collection

Demographic and clinical data was collected onto a validated case report form (CRF) for the study period. Mortality in hospital and at 28 days was collected through chart review and telephone contact as applicable.

### Definitions

Blood culture results from blood samples collected within 24 hours of admission were evaluated. Patients with more than one blood stream pathogen were excluded from analysis. Presenting clinical syndromes were classified based on the primary diagnoses of attending physicians. The clinical syndromes were grouped into acute febrile illness, lower respiratory infection, diarrheal illness, sepsis, septic shock and others [16]. Infection with organ dysfunction (sepsis) was determined in accordance with recent sepsis guidelines; a modified sequential organ failure assessment (SOFA) score (continuous and categorized as ≥2 and <2) within 24 hours of admission was used to define organ dysfunction and is detailed elsewhere [16, 20, 21]. Laboratory values of hemoglobin, hematocrit, platelets, white blood cells (WBC), blood urea nitrogen (BUN), creatinine, albumin, aspartate transaminase (AST), bilirubin, and international normalized ratio (INR) within 24 hours of admission were used for analysis. Recommended antibiotics for treatment of *E*. *coli* bacteraemia were aminoglycosides, third-generation cephalosporins, fluoroquinolones, penicillin plus beta-lactamase inhibitors and carbapenems [22, 23]. Recommended antibiotics for treatment of *B*. *pseudomallei* bacteraemia were ceftazidime, carbapenems and amoxicillin/clavulanic acid [24, 25]. Recommended antibiotics for treatment of *S*. *aureus* bacteraemia were oxacillin, cefazolin, clindamycin, daptomycin and vancomycin [22, 26].

### Statistical analysis

Continuous and categorical variables were compared with Kruskal-Wallis tests and Chi-square tests, respectively. Fisher’s exact test was used when appropriate. The primary outcome was 28-day mortality. We performed survival analyses using Kaplan-Meier methods and Cox proportional hazard models. The multivariable models were developed using purposeful selection [27]. The modified SOFA score was included as a continuous variable in the models. Because of collinearity between empirical antibiotic prior to or during transfer and empirical antibiotic effective within the first day of hospital admission, we conducted a separate analysis including empirical antibiotic within the first day of hospitalization in the multilevel Cox proportional hazard model. To explore the effect of antimicrobial resistance, a sensitivity analysis was also conducted by categorizing receipt of antibiotics into: (a) did not receive any empirical antibiotics recommended for the causative organism, (b) received recommended empirical antibiotic, to which the causative organism was susceptible and (c) received recommended empirical antibiotics, to which the causative organism was resistant. All analyses were performed with STATA 14.2 (StataCorp, College Station, TX, USA). The final data dictionary and database are accessible online (https://doi.org/10.6084/m9.figshare.14257457).

### Results

**Fig 1:**
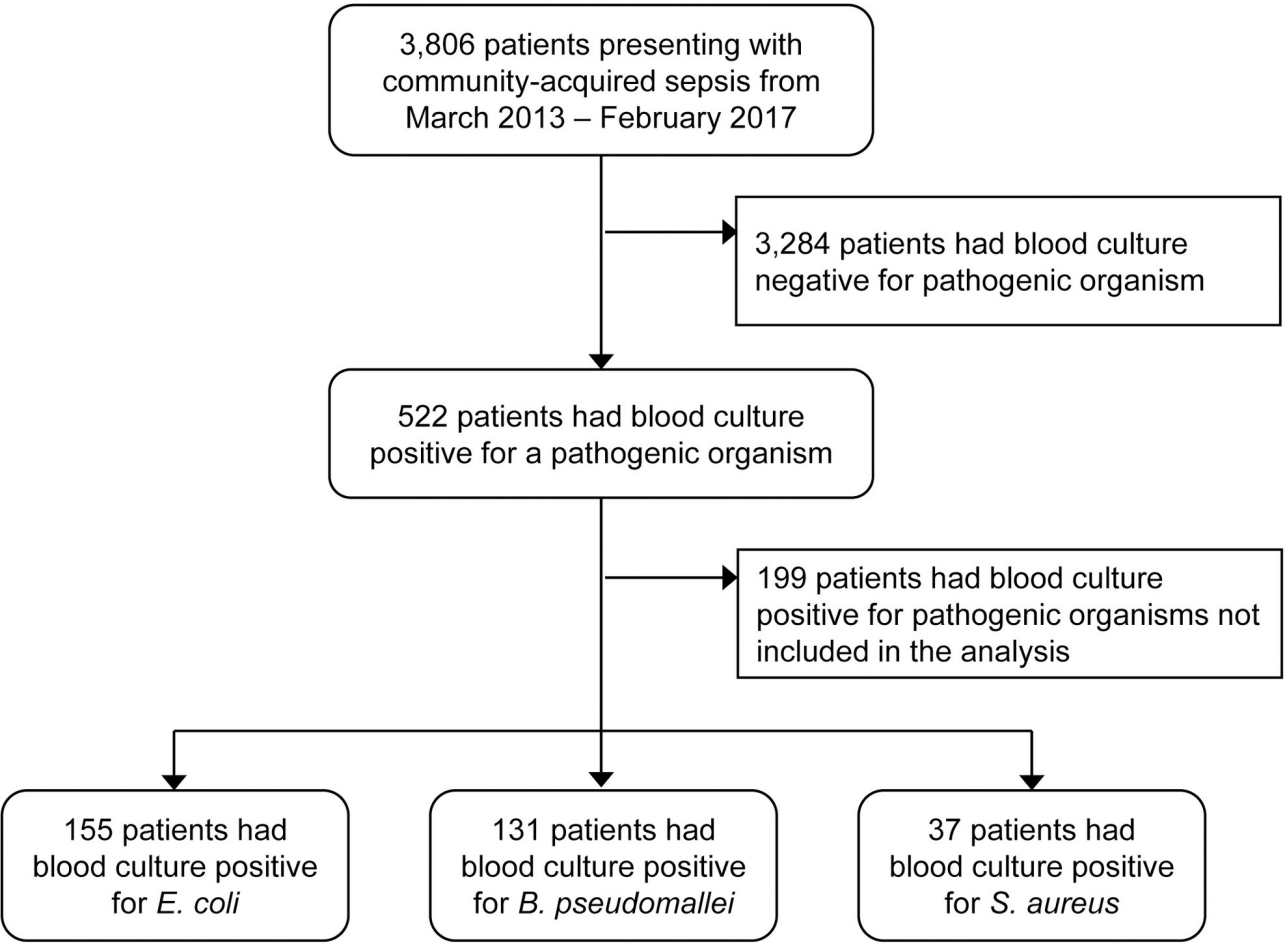
Study flow diagram.

The Ubon-sepsis study prospectively evaluated 3,806 patients presenting with community-acquired sepsis from March 2013-January 2017. A total of 522 (14%) patients had blood collected within 24 hours of admission to the study hospital culture positive for a pathogenic organism. The most common pathogens were *E*. *coli* (n = 155), *B*. *pseudomallei* (n = 131) and *S*. *aureus* (n = 37), and those 323 patients were included for analysis (**Fig 1**).

### Baseline characteristics

The baseline characteristics of 323 patients included in the analysis are described in **Table 1**. The proportion that were male was higher among patients with *B*. *pseudomallei* and *S*. *aureus* bacteraemias (66% and 73%, respectively) compared to those with *E*. *coli* bacteraemia (42%) (p<0.001). The median age was highest among patients with *E*. *coli* bacteraemia (65 years, IQR 57–75 years) and lowest among those with *B*. *pseudomallei* bacteraemia (56 years, IQR 46–66 years) (p<0.001). A known history of diabetes mellitus was more common in patients with *B*. *pseudomallei* bacteraemia (48%) (p = 0.01) and known history of chronic kidney disease was more common in patients with *S*. *aureus* bacteraemia (32%) (p = 0.05). The proportion of patients transferred from other hospitals was highest in patients with *B*. *pseudomallei* bacteraemia (94%), followed by those with *S*. *aureus* (86%) and *E*. *coli* (83%) bacteraemias (p = 0.02). The proportion of patients presenting with lower respiratory infection was highest in patients with *B*. *pseudomallei* bacteraemia (40%), followed by those with *S*. *aureus* (35%) and *E*. *coli* (21%) bactereamia (p = 0.003).

**Table 1:** Baseline clinical characteristics of patients with community-acquired *E*. *coli*, *B*. *pseudomallei* and *S*. *aureus* bacteraemias.

Parameters	*E*. *coli* (n = 155)	*B*. *pseudomallei* (n = 131)	*S*. *aureus* (n = 37)	P value
**Male gender**	65 (42%)	87 (66%)	27 (73%)	<0.001
**Age (years), median (IQR)**	65 (57–75)	56 (46–66)	63 (56–70)	<0.001
**Age group (years)**				
18–40	9 (6%)	15 (11%)	3 (8%)	<0.001
>40–60	40 (26%)	67 (51%)	10 (27%)	
>60–70	51 (33%)	26 (20%)	14 (38%)	
>70	55 (35%)	23 (18%)	10 (27%)	
**Comorbidities:**				
Hypertension	52 (34%)	35 (27%)	13 (35%)	0.39
Diabetes mellitus	48 (31%)	63 (48%)	12 (32%)	0.01
Chronic kidney disease	23 (15%)	24 (18%)	12 (32%)	0.05
Dyslipidemia	12 (8%)	8 (6%)	0 (0%)	0.21
Heart disease	11 (7%)	9 (7%)	4 (11%)	0.70
Chronic lung disease	3 (2%)	9 (7%)	1 (3%)	0.10
Liver disease	10 (6%)	6 (5%)	1 (3%)	0.59
Cerebrovascular disease	8 (5%)	2 (2%)	1 (3%)	0.23
Malignancy	1 (1%)	1 (1%)	2 (5%)	0.05
**Transferred from other hospitals**	129 (83%)	123 (94%)	32 (86%)	0.02
**Duration of symptoms** (days)	2 (1–3)	4 (2–7)	2 (1–4)	<0.001
**Presenting clinical syndromes** *				
Acute febrile illness	48 (31%)	44 (34%)	11 (30%)	0.85
Lower respiratory infection	33 (21%)	52 (40%)	13 (35%)	0.003
Diarrheal illness	15 (10%)	11 (8%)	3 (8%)	0.91
Septic shock	82 (53%)	58 (44%)	14 (38%)	0.15
Sepsis	34 (22%)	25 (19%)	11 (30%)	0.38
Others	14 (9%)	2 (2%)	1 (3%)	0.01

* Patients may have more than one presenting clinical syndrome

On enrollment, there was borderline evidence of higher blood lactate level in patients with *B*. *pseudomallei* bacteraemia (median 3.3 mmol/L [IQR 1.9–6.6 mmol/L]) than in those with *E*. *coli* (median 2.6 mmol/L [IQR 1.6–4.8 mmol/L]) and *S*. *aureus* (median 2.8 mmol/L [IQR 1.7–4.0 mmol/L]) bacteraemias (p = 0.08). The median modified SOFA score was higher in patients with *B*. *pseudomallei* bacteraemia (7 [IQR 5–10]) than in those with *E*. *coli* (6 [IQR 4–8]) and *S*. *aureus* (6 mmol/L [IQR 4–7]) bacteraemias (p = 0.05).

### Empirical antibiotic treatment

Of 284 patients transferred from other hospitals, 225 (79%) received at least one antibiotic prior to or during transfer. Ceftriaxone was the most commonly administered antibiotic (N = 178, 55%), followed by ceftazidime (N = 52, 16%), clindamycin (N = 22, 7%), doxycycline (N = 18, 6%), metronidazole (N = 11, 3%), macrolide drugs (N = 8, 2%), cloxacillin (N = 4, 2%), carbapenem drugs (N = 4, 1%), ciprofloxacin (N = 3, 1%), cefazolin (N = 2, 1%) and aminoglycosides (N = 1, 0.3%). Of 129, 123 and 32 patients with *E*. *coli*, *B*. *pseudomallei* and *S*. *aureus* bacteria transferred from other hospitals, 97 (75%), 31 (25%) and 8 (25%), respectively, received recommended empirical antibiotics for the relevant bacteremia prior to or during transfer (P<0.001).

Of all 323 patients, 315 (98%) received at least one antibiotic within the first day of hospitalization at the study hospital. Of all 155, 131 and 37 patients with *E*. *coli*, *B*. *pseudomallei* and *S*. *aureus* bacteria, 149 (96%), 98 (75%) and 18 (49%), respectively, received recommended antibiotics for the relevant bacteremia given empirically within the first day of hospitalization (p<0.001).

### Clinical outcomes

**Fig 2:**
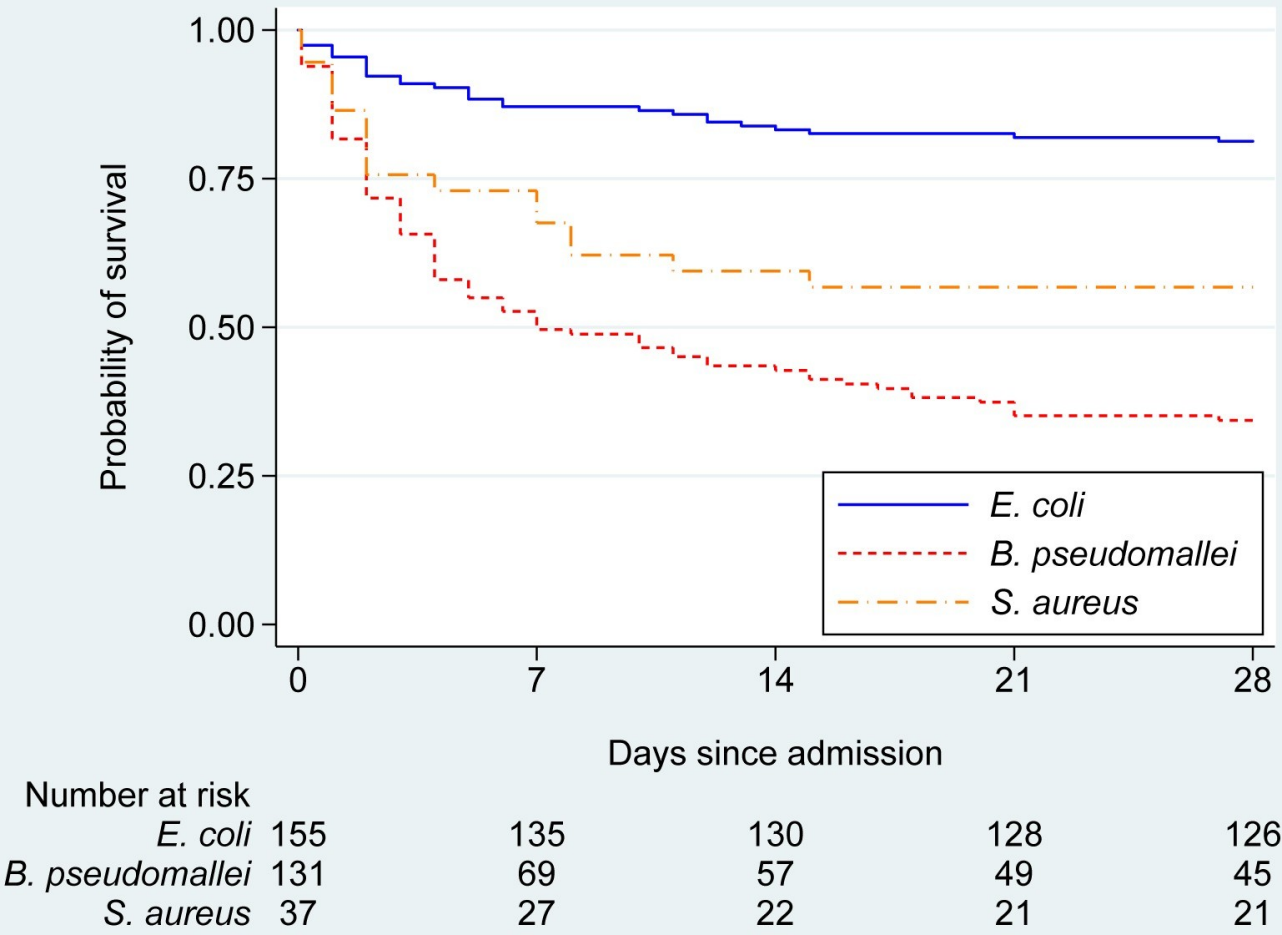
Probability of survival comparing patients with community-acquired *E*. *coli*, *B*. *pseudomallei* and *S*. *aureus* bacteraemias.

A total of 132 deaths occurred in the study period resulting in an overall 28-day mortality of 41% (**Table 2**). Patients with *B*. *pseudomallei* bacteraemia had a 28-day mortality of 66% (87/131) compared with 43% (16/37) for *S*. *aureus* and 19% for *E*. *coli* (29/155) bacteraemias (p<0.001). Among those who died, the median time to death was shortest in the *S*. *aureus* group (2 days (1–8)) but the difference was not statistically significant between groups (p = 0.76) (Table 2). Among those who survived, the median length of stay at the study hospital was greatest in the *B*. *pseudomallei* group (13 days (7–22), p<0.001). Survival curves demonstrated that the majority of deaths occurred within a week of admission (**Fig 2**).

**Table 2:** Day 28 mortality and duration of hospitalization in patients with community acquired bacteremia by pathogen.

Outcomes	*E*. *coli* (n = 155)	*B*. *pseudomallei* (n = 131)	*S*. *aureus* (n = 37)	P value
28-day mortality (n, [%])	29 (19%)	87 (66%)	16 (43%)	<0.001
Time to death (days, median [IQR])*	4 (2–11)	3 (1–7)	2 (1–8)	0.76
Length of hospital stay (days, median [IQR])**	7 (5–12)	13 (7–22)	7 (5–11)	<0.001

* Among those who died within 28 days

** Among those who survived to 28 days

In a multivariable Cox proportional hazard model adjusted for age, sex, transfer status, empirical antibiotics prior to or during transfer, comorbidities and modified SOFA on admission, *B*. *pseudomallei* (adjusted hazard ratio [aHR] 3.78; 95%CI 2.31–6.21) and *S*. *aureus* (aHR 2.72; 95%CI 1.40–5.28) bacteraemias were associated with higher mortality compared to those with *E*. *coli* bacteraemia (p<0.001). Empiric administration of recommended antibiotics for the relevant bacteraemia prior to or during transfer was associated with survival (aHR 0.58; 95%CI 0.38–0.88, p = 0.01) (Table 3). Because of collinearity with empiric administration of recommended antibiotics prior to or during transfer, empiric administration of recommended antibiotics within the first day of hospital admission was not included in the final model. We included empiric administration of recommended antibiotics within the first day of hospital admission in a separate multivariable model and found that this variable was not independently associated with mortality following infection (p = 0.97) (S1 Table).

**Table 3:** Factors associated with 28-day mortality among patients with community acquired bacteraemia using Cox proportional hazards models.

Factors	Crude hazard ratio (95% CI)	P value	Adjusted hazard ratio (95% CI)	P value
**Age group (years)**				
18–40	1.0	0.07	1.0	0.12
>40–60	0.99 (0.54–1.81)		0.93 (0.50–1.73)	
>60–70	0.55 (0.28–1.06)		0.86 (0.43–1.74)	
>70	0.84 (0.45–1.58)		1.50 (0.77–2.95)	
**Male gender**	1.29 (0.91–1.82)	0.16	0.68 (0.47–0.99)	0.04
**Transferred from other hospital**	2.85 (1.33–6.10)	0.007	1.80 (0.80–4.08)	0.16
**Comorbidities (n [%])**				
Diabetes mellitus	1.09 (0.77–1.54)	0.64	0.99 (0.69–1.42)	0.96
Chronic kidney disease	1.18 (0.77–1.79)	0.45	1.32 (0.84–2.07)	0.23
Liver disease	0.68 (0.28–1.66)	0.40	0.60 (0.24–1.53)	0.29
Malignancy	1.19 (0.29–4.81)	0.81	1.44 (0.33–6.19)	0.63
**Modified SOFA score within 24 hours of admission**	1.22 (1.17–1.28)	<0.001	1.25 (1.18–1.32)	<0.001
**Blood culture**				
*Escherichia coli*	1.0	<0.001	1.0	<0.001
*Burkholderia pseudomallei*	4.96 (3.25–7.57)		3.78 (2.31–6.21)	
*Staphylococcus aureus*	2.78 (1.51–5.11)		2.72 (1.40–5.28)	
**Empirical antibiotic recommended for CAB caused by the etiologic organism prior to or during transfer** *	0.65 (0.46–0.94)	0.02	0.58 (0.38–0.88)	0.01

*Recommended antibiotics for treatment of *E*. *coli* bacteraemia were aminoglycosides, third-generation cephalosporins, fluoroquinolones, penicillin plus beta-lactamase inhibitors and carbapenems [22, 23]. Recommended antibiotics for treatment of *B*. *pseudomallei* bacteraemia were ceftazidime, carbapenems and amoxicillin/clavulanic acid [24, 25]. Recommended antibiotics for treatment of *S*. *aureus* bacteraemia were oxacillin, cefazolin, clindamycin, daptomycin and vancomycin [22, 26].

To evaluate the potential effect of antibiotic resistance on the association of empirical antibiotics with outcome, we conducted a sensitivity analysis by categorizing empirical antibiotics based on antimicrobial susceptibility test results of the isolated organisms (S2 and S3 Tables). Of the 155 isolates of *E*. *coli*, 53 (34%) were resistant to 3^rd^ generation cephalosporins and 2 (1%) were resistant to 3^rd^ generation cephalosporins and carbapenem drugs. All of the *B*. *pseudomallei* isolates were susceptible to ceftazidime, amoxicillin/clavulanic acid and carbapenem drugs. Of the 37 isolates of *S*. *aureus*, 4 (11%) were methicillin-resistant *S*. *aureus* (MRSA). In a multivariable model, there was no difference in mortality comparing patients with CAB caused by an organism sensitive to the empirical antibiotic administered prior to or during transfer to patients with CAB caused by an organism resistant to the empirical antibiotic administered prior to or during transfer (aHR 0.60 vs. 0.43, p = 0.52, S2 Table). We also did not observe a difference in mortality when repeating this analysis for empirical antibiotics given within the first day of hospitalization (aHR 0.99 vs. 0.91, p = 0.24, S3 Table). Mortality of patients with CAB caused by *B*. *pseudomallei* was higher than morality in those patients with CAB caused by *S*. *aureus* and *E*. *coli* in all models (S1, S2 and S3 Tables).

## Discussion

In our comparative prospective observational study of hospitalized patients with community-acquired infections in Northeastern Thailand, we demonstrated that CAB with the most prevalent pathogens of *E*. *coli*, *B*. *pseudomallei*, and *S*. *aureus* was associated with a significant 28-day mortality of 41%. Mortality of patients with CAB caused by *B*. *pseudomallei* was higher than those caused by *S*. *aureus* and *E*. *coli*, even after adjusting for transfer status, effectiveness of empirical antibiotics received prior to or during the transfer and presence of organ dysfunction on admission. Most of patients in our setting at a public tertiary-care hospital were transferred from other hospital. We found that transfer from other hospitals was associated with higher mortality [16]. The distance from the referring hospital to the study hospital could be up to 200 kilometers and the travel duration could be up to two to three hours by ambulance. However, receipt of effective empirical antibiotic against the causative pathogen prior to or during transfer was associated with survival. Antimicrobial resistance was not uncommon. The prevalence of 3^rd^ generation cephalosporin resistance among community-acquired *E*. *coli* bacteremia was 35%. Nonetheless, in our setting, we did not observe that patients who had CAB caused by 3^rd^ generation cephalosporin-resistant *E*. *coli* (3GCREC) and received empirical treatment with 3^rd^ generation cephalosporins had higher mortality. We found no evidence to support the use of antibiotics recommended against 3GCREC (such as carbapenem drugs) as the first-line empirical treatment of community-acquired sepsis. Our findings suggest that improving algorithms or rapid diagnostic tests to guide the administration of empirical antibiotic, if possible prior to or during transfer, could improve the outcomes of CAB in LMICs.

As the epidemiology of CAB pathogens varies between regions, it is critical to understand local epidemiology for implementation of optimal therapeutic initiatives. Both *E*. *coli* and *S*. *aureus* are similarly observed as frequent pathogens in CAB cases in both high and low-income settings [4–6, 9, 28–31]. In our study, *B*. *pseudomallei*, the cause of melioidosis, was the second most frequently isolated bloodstream pathogen (16%). Given the environmental predilection of *B*. *pseudomallei* to tropical climates, it is a common CAB pathogen in studies of South/East Asia as well as Northern Australia [32–34]. However, the presence and prevalence of *B*. *pseudomallei* as a pathogen may still be underestimated in many tropical developing countries due to limitations in microbiology laboratory facilities, resources, and expertise [35].

Selecting empiric antimicrobial therapy to cover pathogens commonly associated with CAB remains challenging. We identified that 75% of patients with CAB caused by *E*. *coli* had empiric antibiotics considered effective against *E*. *coli* prior to or during transfer. However, 25% of patients with CAB caused by *B*. *pseudomallei* and *S*. *aureus* had empiric antibiotics considered effective against *B*. *pseudomallei* and *S*. *aureus* prior to or during transfer. The percentage of having empiric antibiotics considered effective against *B*. *pseudomallei* and *S*. *aureus* was similarly low even within the first day of hospitalization at the study hospital. The low antimicrobial coverage of *B*. *pseudomallei* prior to or during transfer may have been due to lack of ceftazidime at district hospitals; however, this issue should not apply to *S*. *aureus* as cloxacillin and clindamycin are fully available in all district hospitals in Thailand suggesting a possibility of under-recognition or limitation of guidelines for antibiotics [36, 37]. The coverage of empirical antibiotics for patients with CAB caused by *S*. *aureus* within the first day of hospitalization in this study was low compared to early effective antibiotic therapy for patients with *S*. *aureus* infection in a previous study [14] (46% vs. 87%) and may have been due to different study designs. In this study, we enrolled only sepsis patients presenting to the hospital within 24 hours of hospital admission, while the previous study included all patients with a culture positive for *S*. *aureus* from any sterile sites and included both community-acquired and hospital-acquired *S*. *aureus* infections [14]. Further studies are required to evaluate and study how to improve the selection of empiric antimicrobial therapy for community-acquired sepsis in different settings in LMICs.

Our study has a number of strengths including the prospective study design, detailed data on organ dysfunction, a large geographic catchment area, extremely high rates of follow-up and minimal missingness of assessment data. Additionally, it is unique in its approach to a comparative assessment of the outcomes following CAB by pathogen group. However, we must consider a number of limitations. Given the resource-limited setting, although clinical assessments were captured, equipment-based testing (beyond point-of-care) was not always completed. Blood cultures were generally taken at a single time-point thus limiting our ability to delineate between transient, intermittent, and continuous bacteremia cases. This may have led to an underestimate of both the incidence and severity of cases. However, given our illness burden and mortality, it is unlikely that patients were misclassified as true infections in this cohort. It is possible that a small proportion of bacteremia cases in our study cohort were hospital acquired but this was unlikely based on our study design, timepoint cutoffs, participants presenting with sepsis, and our restriction to three pathogens that are causes of community-acquired infections. As our mortality assessment was all-cause mortality within 28 days of enrollment, the mortality might reflect other causes of death such as exacerbation of underlying diseases [38]. It is possible that we were still underpowered to account for all potential confounders including baseline socio-demographic and clinical parameters. Also, our sample size was too small to evaluate the specific association between 3^rd^ generation cephalosporin and mortality among patients with methicillin-sensitive *S*. *aureus* bacteremia. Despite the limitations, this is one of the first prospective studies to examine the characteristics and outcomes of CAB by pathogen group in a LMIC setting.

## Conclusion

In our large comparative prospective evaluation of CAB in Northeastern Thailand, we identified that CAB is associated with a significant burden of mortality. CAB caused by *B*. *pseudomallei* and *S*. *aureus* are associated with higher mortality than CAB caused by *E*. *coli* even after adjusting for presence of organ dysfunction on admission and effectiveness of empirical antibiotics received. In our setting, a tertiary-care hospital in a LMIC, most patients with CAB are transferred from other hospitals and administration of recommended empirical antibiotics prior to or during transfer is associated with survival.

## Supporting information

S1 Table Factors associated with 28-day mortality among patients with community acquired bacteraemia. (DOCX)

S2 Table Factors associated with 28-day mortality among patients with community acquired bacteraemia. (DOCX)

S3 Table Factors associated with 28-day mortality among patients with community acquired bacteraemia. (DOCX)
