# Supplementary material for: Comparative clinical characteristics and outcomes of patients with community acquired bacteremia caused by Escherichia coli, Burkholderia pseudomallei and Staphylococcus aureus: A prospective observational study (Ubon-sepsis)
Source: PLoS Negl Trop Dis. 2021 Sep 3;15(9):e0009704. doi: 10.1371/journal.pntd.0009704 (PMC8415581; doi:10.1371/journal.pntd.0009704)
Supplement: S2 Table — (DOCX) [file pntd.0009704.s002.docx]

# Table S2. Factors associated with 28-day mortality among patients with community acquired bacteraemia using Cox proportional hazards models (sensitivity analysis 2)

| **Factors** | **Crude hazard ratio**  **(95% CI)** | **P value** | **Adjusted hazard ratio**  **(95% CI)** | **P value** |
| --- | --- | --- | --- | --- |
| **Age group (years)** |  |  |  |  |
| 18-40 | 1.0 | 0.07 | 1.0 | 0.12 |
| >40-60 | 0.99 (0.54-1.81) |  | 0.93 (0.50-1.73) |  |
| >60-70 | 0.55 (0.28-1.06) |  | 0.86 (0.42-1.73) |  |
| >70 | 0.84 (0.45-1.58) |  | 1.50 (0.77-2.95) |  |
| **Male gender** | 1.29 (0.91-1.82) | 0.16 | 0.69 (0.47-1.00) | 0.05 |
| **Transferred from other hospital** | 2.85 (1.33-6.10) | 0.007 | 1.85 (0.82-4.21) | 0.14 |
| **Comorbidities (n [%])** |  |  |  |  |
| Diabetes mellitus | 1.09 (0.77-1.54) | 0.64 | 0.99 (0.69-1.42) | 0.95 |
| Chronic kidney disease | 1.18 (0.77-1.79) | 0.45 | 1.33 (0.85-2.08) | 0.22 |
| Liver disease | 0.68 (0.28-1.66) | 0.40 | 0.62 (0.24-1.56) | 0.31 |
| Malignancy | 1.19 (0.29-4.81) | 0.81 | 1.48 (0.34-6.42) | 0.60 |
| **Modified SOFA score within 24 hours of admission** | 1.22 (1.17-1.28) | <0.001 | 1.24 (1.18-1.31) | <0.001 |
| **Blood culture** |  |  |  |  |
| *Escherichia coli* | 1.0 | <0.001 | 1.0 | <0.001 |
| *Burkholderia pseudomallei* | 4.96 (3.25-7.57) |  | 3.58 (2.14-5.99) |  |
| *Staphylococcus aureus* | 2.78 (1.51-5.11) |  | 2.57 (1.30-5.07) |  |
| **Recommended antibiotic given empirically prior to or during transfer** |  |  |  |  |
| No | 1.0 | <0.001 | 1.0 | 0.03 |
| Yes and isolated organism susceptible to the empirical antibiotic given | 0.86 (0.59-1.25) |  | 0.60 (0.39-0.92)***** |  |
| Yes and isolated organism resistant to the empirical antibiotic given | 0.22 (0.09-0.55) |  | 0.43 (0.16-1.18)***** |  |

* In the multivariable model, no difference was observed between the isolated organisms resistant and not resistant to the empirical antibiotic received (p=0.52)
