# Supplementary material for: Comparative clinical characteristics and outcomes of patients with community acquired bacteremia caused by Escherichia coli, Burkholderia pseudomallei and Staphylococcus aureus: A prospective observational study (Ubon-sepsis)
Source: PLoS Negl Trop Dis. 2021 Sep 3;15(9):e0009704. doi: 10.1371/journal.pntd.0009704 (PMC8415581; doi:10.1371/journal.pntd.0009704)
Supplement: S3 Table — (DOCX) [file pntd.0009704.s003.docx]

# Table S3. Factors associated with 28-day mortality among patients with community acquired bacteraemia using Cox proportional hazards models (sensitivity analysis 3)

| **Factors** | **Crude hazard ratio**  **(95% CI)** | **P value** | **Adjusted hazard ratio**  **(95% CI)** | **P value** |
| --- | --- | --- | --- | --- |
| **Age group (years)** |  |  |  |  |
| 18-40 | 1.0 | 0.07 | 1.0 | 0.21 |
| >40-60 | 0.99 (0.54-1.81) |  | 0.93 (0.49-1.74) |  |
| >60-70 | 0.55 (0.28-1.06) |  | 0.88 (0.43-1.79) |  |
| >70 | 0.84 (0.45-1.58) |  | 1.42 (0.72-2.82) |  |
| **Male gender** | 1.29 (0.91-1.82) | 0.16 | 0.75 (0.52-1.09) | 0.14 |
| **Transferred from other hospital** | 2.85 (1.33-6.10) | 0.007 | 1.47 (0.66-3.29) | 0.35 |
| **Comorbidities (n [%])** |  |  |  |  |
| Diabetes mellitus | 1.09 (0.77-1.54) | 0.64 | 0.97 (0.67-1.39) | 0.86 |
| Chronic kidney disease | 1.18 (0.77-1.79) | 0.45 | 1.36 (0.86-2.14) | 0.19 |
| Liver disease | 0.68 (0.28-1.66) | 0.40 | 0.60 (0.23-1.52) | 0.28 |
| Malignancy | 1.19 (0.29-4.81) | 0.81 | 1.41 (0.32-6.28) | 0.64 |
| **Modified SOFA score within 24 hours of admission** | 1.22 (1.17-1.28) | <0.001 | 1.22 (1.16-1.28) | <0.001 |
| **Blood culture positive** |  |  |  |  |
| *Escherichia coli* | 1.0 | <0.001 | 1.0 | <0.001 |
| *Burkholderia pseudomallei* | 4.96 (3.25-7.57) |  | 4.57 (2.76-7.57) |  |
| *Staphylococcus aureus* | 2.78 (1.51-5.11) |  | 3.21 (1.60-6.46) |  |
| **Recommended antibiotic given empirically within the first day of hospitalization** |  |  |  |  |
| No | 1.0 | 0.003 | 1.0 | 0.98 |
| Yes and isolated organism susceptible to the empirical antibiotic given | 0.96 (0.63-1.49) |  | 0.99 (0.60-1.64)* |  |
| Yes and isolated organism resistant to the empirical antibiotic given | 0.32 (0.14-0.74) |  | 0.91 (0.35-2.36)* |  |

* In the multivariable model, difference was not observed between the isolate organisms resistant and not resistant to the empirical antibiotic received (p=0.24)
